# Impulsivity and overeating: a closer look at the subscales of the Barratt Impulsiveness Scale

**DOI:** 10.3389/fpsyg.2013.00177

**Published:** 2013-04-10

**Authors:** Adrian Meule

**Affiliations:** Department of Psychology I, University of WürzburgWürzburg, Germany

Impulsivity can be defined as a predisposition toward rapid, unplanned reactions to internal or external stimuli regardless of negative consequences of these reactions for the impulsive individual or for others (Moeller et al., 2001). It is a multifaceted construct and there is a range of methods available for its measurement. Two of the most often used methods are self-report instruments and behavioral tasks (e.g., go/no-go tasks and delay discounting tasks). Self-reported impulsivity is positively correlated with impulsive reactions in behavioral measures, yet correlations are often weak and inconsistent (Logan et al., 1997; Lijffijt et al., 2004; Enticott et al., 2006; Reynolds et al., 2006; Cyders and Coskunpinar, 2011). It is assumed that self-report questionnaires represent impulsivity as a stable trait while behavioral tasks are subject to state-dependent variations. Nonetheless, both self-report and behavioral measures indicate that high impulsivity is a risk factor for a range of maladaptive behaviors, including over- or binge eating (Guerrieri et al., 2008; Waxman, 2009).

Beyond the fact that self-report and behavioral measures seem to capture different aspects of impulsivity, conceptualizations also vary between the different self-report instruments. For instance, two of the most widely used impulsivity questionnaires are the *UPPS Impulsive Behavior Scale* (Whiteside and Lynam, 2001) and the *Barratt Impulsiveness Scale* (BIS-11, Patton et al., 1995). The UPPS assesses impulsivity on the subscales *urgency* (acting rashly under conditions of negative affect), *lack of premeditation* (difficulty in thinking and reflecting on consequences of an act), *lack of perseverance* (inability to remain focused on a task), and *sensation seeking* (tendency and openness to try and enjoy exciting or dangerous activities). The BIS-11 assesses impulsivity on the subscales *attentional impulsivity* (inability to focus attention or concentrate), *motor impulsivity* (acting without thinking), and *non-planning impulsivity* (lack of future orientation or forethought). Both questionnaires are highly correlated with each other (*r* = 0.67), but correlations between their subscales are only weak and inconsistent, supporting the notion that both measures cover different aspects of impulsivity (Meule et al., 2011). Beyond using the UPPS total score, relationships between UPPS subscales and eating behavior have been investigated and it has been found that urgency in particular is predictive for eating problems, e.g., binge eating (Fischer et al., 2003, 2008; Smith et al., 2007; Mobbs et al., 2008; Manwaring et al., 2011; Dir et al., 2013). To date, similar clear-cut results for the BIS-11 are missing. Although it is widely used, most studies only use its total score for analysis. In this brief opinion piece I would like to advocate the use of BIS-subscales. That is, researchers may benefit from examining relationships between BIS-subscales and eating behavior in greater detail.

Only a few studies have done this as yet. For instance, differential relationships between subscales of the BIS-11 and eating disorder symptomatology have been found in clinical samples. Patients with binge eating disorder had higher scores on the motor impulsivity subscale compared to healthy controls, but did not differ on the other two subscales (Nasser et al., 2004). Two studies compared scores on the BIS-11 between patients with bulimia nervosa (BN), anorexia nervosa—binge/purge type (AN-BP), anorexia nervosa—restrictive type (AN-R), and healthy controls. In a first study by Rosval and colleagues (2006), eating disorder groups did not differ from each other on the attentional impulsivity subscale, but all had higher scores than controls. With regard to motor impulsivity, the two groups with binge eating behavior (BN and AN-BP) had higher scores than both the AN-R group and controls. The BN group also had higher scores on non-planning impulsivity than both the AN-BP and AN-R group, but did not differ from controls (Rosval et al., 2006). In a second study (Claes et al., 2006), the two groups with binge eating (BN and AN-BP) reported higher attentional impulsivity compared to controls. With regard to motor impulsivity, AN-BP, BN, and controls had higher scores than AN-R. The BN group and controls also had higher scores on non-planning impulsivity than AN-R (Claes et al., 2006). In sum, it appears that eating disorder patients with binge eating behaviors have higher BIS-11 scores, particularly on its motor and attentional impulsivity subscales, compared to patients with restrictive eating behavior and controls.

Studies investigating non-clinical samples also revealed differential associations between BIS-11 subscales and various measures of eating behavior. For example, Lyke and Spinella (2004) examined the associations between the BIS-11 and the *Eating Inventory* (formerly *Three-Factor Eating Questionnaire*, Stunkard and Messick, 1985). A small positive correlation was found between the *hunger* subscale and attentional impulsivity. Furthermore, both attentional and motor impulsivity were correlated with *disinhibition* (Lyke and Spinella, 2004). There was a small positive association between non-planning impulsivity and rigid control of eating behavior in female, but not male, students (Timko and Perone, 2005).

In a recent study by van Koningsbruggen and colleagues (2013), only the attentional impulsivity subscale of the BIS-11, but not the other two subscales, was positively correlated with the *concern for dieting* subscale of the *Restraint Scale* (Herman and Mack, 1975). In addition, both attentional and non-planning impulsivity were negatively related to self-perceived dieting success. With regard to body-mass-index (BMI), there was a positive correlation with the motor impulsivity subscale (van Koningsbruggen et al., 2013). In a study by Nolan (2012), female students were asked to rate the pleasantness of eating different foods on a visual analog scale and to complete the BIS-11. Only the attentional impulsivity subscale, but not the other two subscales, was positively correlated with the pleasantness of eating high-calorie foods like French fries, pasta, and pizza. No correlations between the BIS-11 and BMI could be found (Nolan, 2012). In a sample of pathological gamblers (von Ranson et al., 2013), only the attentional subscale of the BIS-11, but not the other two subscales, was positively correlated with the *eating concern* subscale of the *Eating Disorder Examination—Questionnaire* (Fairburn and Beglin, 1994).

While the BIS-11 contains 30 items, Spinella (2007) developed a short form of the BIS-11 which consists of 15 items only (BIS-15). The three-factorial structure of the long version could also be found using the short form and each subscale contains five items (Spinella, 2007; Meule et al., 2011). Scores of the short form are highly correlated with the full version (*r* = 0.94, Spinella, 2007).

In a range of studies, positive correlations could be found between the BIS-15 and various constructs that are related to overeating (Table 1), e.g., frequent and intense experiences of food cravings (*Food Cravings Questionnaire—Trait*, Cepeda-Benito et al., 2000), emotional eating (*Mood Eating Scale*, Jackson and Hawkins, 1980), night eating (*Night Eating Questionnaire*, Allison et al., 2008), low dieting success (*Perceived Self-Regulatory Success in Dieting Scale*, Meule et al., 2012e), restrained eating (*Restraint Scale*, Herman and Mack, 1975), and food addiction symptomatology (*Yale Food Addiction Scale*, Gearhardt et al., 2009). It appears that associations with those measures were particularly observed for the attentional impulsivity subscale while there were no or only negligible relationships with the other subscales in both student samples and severely obese candidates for bariatric surgery (Table 1). Notably, BMI was rarely correlated with the BIS-15 and in some instances correlations were even negative (Table 1).

**Table 1:** **Correlations between the *Barratt Impulsiveness Scale—short form*, body-mass-index, and various self-report measures associated with overeating**.

	**Barratt Impulsiveness Scale—short form**			
	**Attentional**	**Motor**	**Non-planning**	**Total score**
**STUDENT SAMPLES**				
Body-mass-index (kg/m^2^)^1^^,^ ^2^^,^ ^3^^,^ ^4^^,^ ^5^^,^ ^6^	−0.03 to 0.19	−0.15 to 0.11	−0.10 to 0.20	−0.09 to 0.15
Food Cravings Questionnaire—Trait^2^^,^ ^4^^,^ ^5^^,^ ^6^	0.26 to **0.38**	0.07 to 0.23	−0.05 to 0.08	0.13 to 0.28
Mood Eating Scale^4^	**0.30**	0.18	0.03	0.23
Night Eating Questionnaire^4^	**0.32**	0.15	0.11	0.26
Perceived Self-Regulatory Success in Dieting^2^^,^ ^3^^,^ ^5^^,^ ^6^	−**0.36** to −0.20	0.01 to 0.11	−0.04 to 0.04	−0.14 to −0.06
Restraint Scale^3^^,^ ^5^^,^ ^6^	0.22 to 0.26	−0.01 to 0.12	−0.13 to 0.05	0.02 to 0.20
Yale Food Addiction Scale—symptom count^1^^,^ ^2^^,^ ^3^^,^ ^6^	0.22 to **0.34**	0.06 to 0.15	0.00 to 0.13	0.12 to 0.26
**BARIATRIC PATIENTS^7^**
Body-mass-index (kg/m^2^)	−0.23	−0.03	−0.24	−0.22
Eating Disorder Examination—Questionnaire—objective binge episodes	**0.38**	0.04	0.10	0.23
Food Cravings Questionnaire—Trait	**0.45**	0.10	0.09	0.27
Yale Food Addiction Scale—symptom count	**0.39**	0.11	0.11	0.26

*Notes: Numbers in boldface indicate correlations ≥0.3. Note that p-values are not reported because of different sample sizes*.

1 *(Meule et al., 2012f) (n = 752)*,

2 *(Meule et al., 2012c) (n = 616)*,

3 *(Meule et al., 2012d) (n = 50)*,

4 *(Meule et al., 2012a) (n = 729)*,

5 *unpublished data (n = 55)*,

6 *unpublished data (n = 51)*,

7 *(Meule et al., 2012b) (n = 81)*.

Up to now, only one other research group has examined relations between eating behavior measures and BIS-15 subscales. In this study (Hou et al., 2011), the attentional and motor subscale of the BIS-15, but not the non-planning subscale, were positively correlated with the *external eating* subscale of the *Dutch Eating Behavior Questionnaire* (van Strien et al., 1986) and with attentional bias toward high-calorie foods as measured with a visual probe task. In addition, partial correlations revealed that attentional impulsivity mediated the association between external eating and attentional bias (Hou et al., 2011).

In sum, it appears that attentional impulsivity is most consistently related to various measures that are associated with overeating. Positive, but less consistent, relationships can also be found with motor impulsivity, particularly in patients with binge eating behavior. Non-planning impulsivity seems to be only weakly related to overeating. Neither subscale seems to be consistently correlated with BMI, which may be due to the fact that BMI is influenced by many factors other than eating behavior. Future research needs to address the question why attentional impulsivity in particular has such a prominent role in overeating. First evidence suggests that attentional impulsivity may increase the susceptibility that highly palatable food-cues attract attention and trigger eating behavior (Hou et al., 2011). The exact mechanisms, however, are yet to be determined. For example, food-cue induced overeating may be the result of overwhelming, reward-related, bottom-up processes or deficient inhibitory, top-down control mechanisms, or both (Appelhans, 2009; Heatherton and Wagner, 2011). In line with this, it may be that there are also interactive effects of BIS-subscales on overeating which have not been considered in past research. For example, high attentional impulsivity may be related to moderate overeating through reward-sensitive mechanisms (Nolan, 2012), but may be particularly crucial in combination with high motor impulsivity, indicating low inhibitory control. Thus, the use of the BIS-subscales and their interactions has the potential to contribute to the understanding of mechanisms underlying overeating.
